# Phase I study of BIBF 1120 with docetaxel and prednisone in metastatic chemo-naive hormone-refractory prostate cancer patients

**DOI:** 10.1038/bjc.2011.440

**Published:** 2011-10-25

**Authors:** G Bousquet, J Alexandre, C Le Tourneau, F Goldwasser, S Faivre, H de Mont-Serrat, R Kaiser, J L Misset, E Raymond

**Affiliations:** 1 Department of Oncology, APHP – Saint-Louis Hospital, Paris 75010, France; 2 Department of Oncology, APHP – Cochin Hospital, Paris 75005, France; 3 Department of Oncology, Service Inter Hospitalier de Cancérologie (SIHC), APHP – Beaujon University Hospital, 100 Boulevard du General Leclerc, Clichy Cedex 92118, France; 4 Boehringer Ingelheim, Reims Cedex 51060, France; 5 Boehringer Ingelheim Pharma GmbH & Co. KG, Biberach an der Riss 88397, Germany

**Keywords:** phase I, multi-kinase inhibitor, prostate cancer

## Abstract

**Background:**

BIBF 1120 is an oral, potent, tyrosine kinase inhibitor that simultaneously targets vascular endothelial growth factor receptors 1–3, platelet-derived growth factor receptors *α* and *β*, and fibroblast growth factor receptors 1–3, as well as FLT3 and Src. Currently, the molecule is in phase III development for second-line non-small cell lung cancer and first-line ovarian cancer patients.

**Methods:**

This phase I dose-escalation study assessed the safety and maximum tolerated dose of continuous daily treatment with BIBF 1120 plus standard-dose docetaxel (75 mg m^−2^, every 3 weeks) and prednisone (5 mg BID) in patients with metastatic, chemo-naive, hormone-refractory prostate cancer (HRPC). Secondary objectives were characterisation of BIBF 1120 and docetaxel pharmacokinetics (PK), and preliminary antitumour activity.

**Results:**

Patients received BIBF 1120 100 mg BID (*n*=3), 150 mg BID (*n*=3), 200 mg BID (*n*=3), and 250 mg BID (*n*=12). The most frequent drug-related adverse events were diarrhoea (71.4%), asthenia (61.9%), nausea (28.6%), vomiting (28.6%), and alopecia (23.8%). The maximum tolerated dose was 250 mg BID of BIBF 1120. Overall, reversible grade 3/4 liver enzyme elevations occurred in six of twelve patients at this dose level. Among 19 assessable patients, 13 (68.4%) showed a ⩾50% reduction in prostate serum antigen levels from baseline and among 6 evaluable patients with measurable lesions 1 patient experienced a partial response by Response Evaluation Criteria In Solid Tumours criteria. Pharmacokinetic analysis showed no interactions between BIBF 1120 and docetaxel/prednisone.

**Conclusion:**

Based on the overall safety profile, 200 mg BID was the recommended dose for the combination of BIBF 1120 with the standard dose of 75 mg m^−2^ of docetaxel and prednisone that might be further investigated in HRPC patients. This combination was well tolerated, with preliminary signs of efficacy and no indication of PK interaction between BIBF 1120 and docetaxel.

BIBF 1120 is an orally administered indolinone derivative and a potent multi-kinase receptor inhibitor of vascular endothelial growth factor receptor (VEGFR)1, 2, and 3, platelet-derived growth factor receptor (PDGFR)*α* and *β*, and fibroblast growth factor receptor (FGFR)1, 2, and 3, as well as FLT3 and Src. *In vivo*, BIBF 1120 demonstrates potent antitumour activity against human prostate cancer xenografts in athymic mice. Moreover, BIBF 1120 exhibits a sustained inhibition of receptor activation demonstrated by its ability to block VEGFR activation after a 1-h exposure for >32 h (Hilberg *et al*, 2008). In a phase I monotherapy study in patients with advanced, heavily pretreated malignancies, BIBF 1120 showed encouraging antitumour activity and a safety toxicity profile mainly consisting of mild-to-moderate gastrointestinal adverse events (AEs; Mross *et al*, 2010). No cases of hand–foot syndrome, haematological AEs, or severe hypertension were observed. This study also revealed that administration of BIBF 1120 250 mg BID permitted increased drug exposure without additional toxicity. Another phase I study demonstrated that BIBF 1120 200 mg BID can be safely combined with standard doses of paclitaxel and carboplatin (du Bois *et al*, 2010). The observed AE profile was similar to that observed with BIBF 1120 monotherapy, except for chemotherapy-related toxicities. Furthermore, several phase II monotherapy trials have also reported promising signs of efficacy in patients with advanced non-small cell lung cancer (NSCLC) and ovarian cancer (Ledermann *et al*, 2011; Reck *et al*, 2011). Three phase III studies are currently ongoing to elucidate the efficacy of BIBF 1120 in second-line NSCLC (LUME Lung 1 and LUME Lung 2) and ovarian cancer patients (LUME Ovar-1).

Prostate cancer is the second most common cancer in the world and a leading cause of mortality (Coleman *et al*, 2008). Hormone therapy with androgen suppression is the standard treatment for patients with first-line metastatic disease. In hormone-refractory prostate cancer (HRPC), docetaxel with corticosteroid is the only registered chemotherapy associated with a survival advantage, conferring a median survival of <20 months (Petrylak *et al*, 2004; Tannock *et al*, 2004; Berthold *et al*, 2008). After progression on this regimen, there is no standard care procedure, and most treatments offer a response of <20% (Rodney *et al*, 2006; Rosenberg *et al*, 2007). In recent years, several drugs have been evaluated in association with docetaxel but without convincing results (Attia *et al*, 2008; Machiels *et al*, 2008).

Angiogenesis is a mechanism that enables tumours to grow beyond 1–2 mm in diameter (Folkman, 1990). Preclinical data showing increased micro-vessel density in prostate cancer support experimental strategies to target the VEGF and VEGFR pathways (Strohmeyer *et al*, 2004). Several antiangiogenic agents blocking either VEGF or VEGFR have been tested in advanced prostate cancer but none of them have so far demonstrated activity as single agents (Aragon-Ching *et al*, 2010). However, combination of VEGFR2 tyrosine kinase inhibitors may potentiate the effects of docetaxel in prostate cancer cells (Monteverde *et al*, 2011) and available preclinical data suggest that BIBF 1120 in combination with chemotherapy may enhance the activity of cytotoxic agents (Ulahannan and Brahmer, 2011). Although the specific mechanisms underlying the additive or synergistic effects of BIBF 1120 in combination with docetaxel have not yet been fully elucidated, changes in tumour vasculature due to the antiangiogenic effects of BIBF 1120 may facilitate the local delivery of chemotherapy. In addition, BIBF 1120 may also counteract ABC-mediated multi-drug resistance frequently observed during treatments with taxanes (Xiang *et al*, 2011). Therefore, combining BIBF 1120 with standard doses of docetaxel and prednisone as first-line treatment for patients with HRPC was considered as an interesting approach. Besides combining two different modes of action, clinical trials to date report no haematological AEs following BIBF 1120 treatment. This study aims to determine whether BIBF 1120 can be combined with docetaxel and prednisone in this population without compromising treatment safety, pharmacokinetics (PK), or efficacy.

## Patients and methods

This phase I dose-escalation study was approved by the French National Ethics Committee and was conducted in accordance with the Declaration of Helsinki Principles and Good Clinical Practice. A signed informed consent was required for each patient.

### Patient selection

Patients in this study were required to have histologically proven metastatic prostate adenocarcinoma that had continued to progress following hormonal therapy. Such progression was defined as a prostrate serum antigen (PSA) increase of >5 ng ml^−1^ on two occasions despite castrate levels of testosterone, progressive measurable disease according to Response Evaluation Criteria In Solid Tumours (RECIST) criteria and/or progressive bone metastasis indicated by new lesions detected on a bone scan. Patients had a life expectancy of at least 3 months and a World Health Organization (WHO) performance status (PS) ⩽2. No prior treatment for HRPC was permitted, including chemotherapy, biologic response modifier therapy, or any investigational drug. In addition, patients had no major injury or surgery for 4 weeks before the treatment and no prior radiation therapy superior to 30% of the medullar volume. Requirements for study entry included: adequate hepatic function, defined as total bilirubin less than the upper limit of normal (ULN) and transaminases <1.5 × ULN; adequate renal function with serum creatinine 132.6 *μ*mol l^−1^ and adequate bone marrow function with absolute neutrophilic count >1500 per *μ*l; and platelet count >100 000 per *μ*l and haemoglobin >8 mg dl^−1^. Patients with a history of drug addiction or alcoholism, or a requirement for anticoagulation or heparinisation treatment were excluded. Patients were also excluded if they had brain metastasis, a recent history (within the last 6 months) of stroke, angina pectoris, ischaemic cardiomyopathy, cerebral ischaemia or arteritis, or a recent haemorrhagic or evolutive thrombotic event. Gastrointestinal abnormalities that would interfere with intake or absorption of the study drug were also treated as criteria for patient exclusion. These included any requirements for intravenous (IV) alimentation, prior surgical procedures affecting absorption, treatment for peptic ulcers within the last 6 months or active gastrointestinal bleeding unrelated to cancer or malabsorption syndromes.

### Treatment administration

On days 2–21 (21-day cycle), patients took an oral dose of BIBF 1120 twice daily. Patients were instructed to swallow BIBF 1120 with water at the same time every day to ensure a dose interval of ∼12 h. On day 2 of treatment cycles (TCs) 1 and 2, patients only took the morning dose of BIBF 1120, omitting the evening dose to allow PK evaluation. All patients were premedicated with oral corticosteroids (dexamethasone 8 mg per os or methylprednisolone 32 mg per os, at 12 h and 1 h before docetaxel infusion) and subsequently took 5 mg prednisone oral BID throughout the trial. On day 1 of each TC, patients received an IV infusion of 75 mg m^−2^ docetaxel administered over 1 h.

Patients were treated with the combination therapy for a maximum of six cycles and those with no signs of disease progression were offered BIBF 1120 monotherapy at the previously tolerated dose level until unacceptable toxicity or disease progression. Patients who remained on treatment at the end of the trial went on to receive further treatment as part of an extension study.

### Dose escalation and dose-limiting toxicities

Based on prior clinical experience, the starting dose of BIBF 1120 in TC 1 was 100 mg BID; doses were escalated in 50 mg increments until the occurrence of a dose-limiting toxicity (DLT). Recognised DLTs included the occurrence of non-haematological related toxicity grade ⩾3 with the exception of alopecia, nail modifications, acute nausea or vomiting and isolated *γ*-glutamyl transpeptidase elevations. Alternatively, the occurrence of uncomplicated grade 4 neutropenia for >7 days, neutropenia grade ⩾3 associated with fever ⩾38.5 °C, or grade 4 thrombopenia or grade 3 thrombopenia associated with bleeding in any cycle beyond TC 1, was defined as a DLT. In addition, a DLT was declared if BIBF 1120 treatment could not be resumed within 14 days of stopping due to treatment-related toxicity. No intrapatient dose escalation was allowed. When one out of three patients at a particular dose level during TC 1 experienced a DLT, an additional three patients were enrolled onto this dosage group. The maximum tolerated dose (MTD) was defined as the dose at which less than two out of six patients experienced a DLT in TC 1.

### PK and pharmacodynamic analysis

Peripheral blood was collected on days 1, 2, 3, 8, and 15 of TCs 1 and 2, and on days 2 and 15 of TCs 3–6 to perform the PK/pharmacodynamic (PD) analysis. Plasma concentrations of BIBF 1120 were determined after the first dose over the time interval 0–24 h on day 2 of TCs 1 and 2 to investigate single-dose PK characteristics of BIBF 1120 on the day after administration of single doses of docetaxel. Levels of BIBF 1120 were also determined on days 8 and 15 of TCs 1 and 2 to investigate steady-state levels.

Plasma concentrations of BIBF 1120 and docetaxel were analysed by a validated method using high-performance liquid chromatography coupled with tandem mass spectrometry (HPLC-MS/MS) in the Department of Drug Metabolism and Pharmacokinetics (Boehringer Ingelheim Pharma GmbH & Co KG, Biberach, Germany). The assay comprises sample clean-up by automated solid-phase extraction in a 96-well plate format. Chromatography was achieved on an analytical C18 reversed phase HPLC column with gradient elution. The substance was detected and quantified by HPLC-MS/MS using electrospray ionisation in the positive ion mode. Docetaxel was analysed by HPLC-MS/MS using paclitaxel as an internal standard. The assay comprises sample clean-up by liquid–liquid extraction and chromatography on an analytical C18 reversed phase HPLC column with isocratic elution. The detection and quantification of the substance was comparable to the one used for BIBF 1120. The lower limit of quantification for BIBF 1120 and metabolites was 0.5 ng ml^−1^ plasma, using a plasma volume of 200 *μ*l. For docetaxel, the lower limit was 2.5 ng ml^−1^ plasma, using a plasma volume of 100 *μ*l.

The calculated parameters were plasmatic peak concentrations following the first dose (*C*_max_), half-life time (*t*_1/2_), area under the plasma concentration–time curve (AUC_t_), apparent clearance after oral administration, and apparent volume of distribution during the terminal phase.

### Safety analysis and evaluation of response

All patients who received at least one dose of BIBF 1120 or docetaxel were assessed during the safety analysis. Intensity of AEs was graded according to Common Terminology Criteria for Adverse Events (CTCAE) version 3.0. Objective response was defined as a PSA decline ⩾50% from the baseline value over two consecutive courses and/or tumour response according to RECIST criteria (Therasse *et al*, 2000).

## Results

### General

The study was performed in three centres in France from November 2005 to April 2007. A total of 23 patients were recruited, of which 21 patients received at least one cycle of BIBF 1120 (20 days) at doses of 100–250 mg BID, and 2 patients failed screening. The median patient age was 68 years (range 58–79 years) and WHO PS was 0 in 76.2% of patients, and 1 in 23.8% of patients (Table 1). The median time between metastatic diagnosis and the inclusion in the study was 2.7 years (range 0.5–70.6 months). The predominant metastases were in bone (47.6%) and the mean PSA at inclusion in the study was 108.5 ng ml^−1^, ranging from 3 to 1521 ng ml^−1^.

### Safety

As shown in Table 2 presenting the frequency of patients with drug-related AEs, three patients were treated at 100, 150, and 200 mg BID BIBF 1120 dose levels, respectively, and 12 patients at 250 mg BID of BIBF 1120.

During TC 1, the most frequent drug-related AEs were diarrhoea (71.4%), asthenia (61.9%), nausea (28.6%), vomiting (28.6%), and alopecia (23.8%). Overall, the severity of AEs during TC 1 was grade 1 in six patients (28.6%), grade 2 in six patients (28.6%), grade 3 in five patients (23.8%), and grade 4 in three patients (14.3%). Neutropenia was the only grade 4 toxicity and was considered to be related to docetaxel. Grade 3 events were only observed for the patients receiving the 200 and 250 mg BID doses of BIBF 1120. One patient receiving 200 mg BID experienced grade 3 diarrhoea.

Throughout the study period, no patients experienced DLTs at dose levels <250 mg BID of BIBF 1120, allowing escalation to the maximum planned dose of 250 mg BID. At this dose level, overall two out of twelve patients had DLTs consisting of CTCAE grade 3 liver enzyme elevations (*γ*-glutamyltransferase, alanine transaminase, aspartate aminotransferase); one patient within the first cycle and another patient during the later TCs. However, increase of transaminase of CTCAE grade 3 or 4 based on laboratory values was observed in six of twelve patients treated at this dose level during any TC (data not presented). The majority of patients experiencing elevations in hepatic enzymes recovered even when treatment continued unchanged.

The most frequently reported AEs over all TCs and doses, irrespective of relatedness, were asthenia (95.2%), diarrhoea (85.7%), nausea (28.6%), and alopecia (23.8%). Common Grade 3/4 AEs included neutropenia, leucopenia and diarrhoea, most of which were seen in the 250 mg BID dosing group.

All patients completed the six TCs, with three patients requiring dose modification. A patient in the 100-mg BID dose group received only docetaxel with no administration of BIBF 1120 during the sixth course, and two patients in the 250-mg BID group required dose reductions of BIBF 1120 after TC 1 due to hepatic enzyme level elevations. Overall, 16 patients with continuing clinical benefit were entered into the rollover study of BIBF 1120 monotherapy.

### Pharmacokinetics

**Figure 1:**
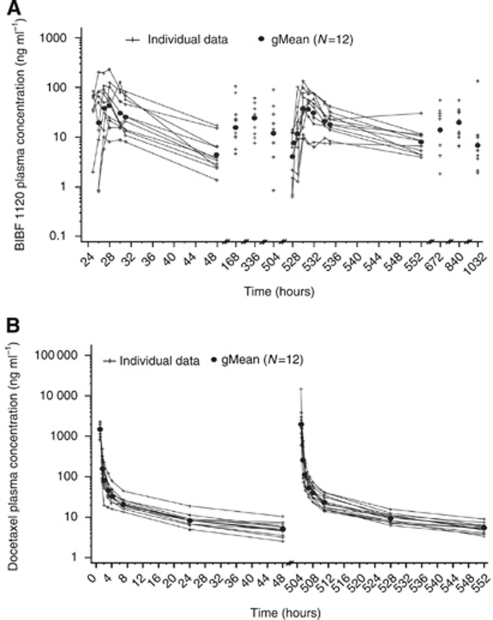
(**A**) Pharmacokinetic profiles of BIBF 1120 following oral administration of 250 mg BIBF 1120 BID dose during the six cycles of treatment (semi-log scale). (**B**) Pharmacokinetic profiles of docetaxel after intravenous administration of 75 mg m^−2^ over 1 h on day 1 of treatment cycles 1 and 2 of patients receiving 250 mg BIBF 1120 BID (semi-log scale).

The main PK parameters of BIBF 1120 and docetaxel are shown in Tables 3 and 4. Following oral administration of 250 mg BIBF 1120 BID dose, time from dosing to peak concentration (*t*_max_) was observed after 3 h, with a high interpatient variability. The individual and geometric mean (gMean) *C*_max_ value was 65.0 ng ml^−1^ on day 2 of TC 1 and 45.9 ng ml^−1^ on day 2 of TC 2 (Figure 1A). On day 2 of TC 1 the gMean AUC_0−24_ value for BIBF 1120 was 454 ng h ml^−1^. The gMean *t*_1/2_ following oral dosing was 7.03 h on day 2 of TC 1 and 15.4 h on day 2 of TC 2. Steady state was reached after 7 days of BIBF 1120 BID dosing. There was no sign of a systematic increase or decrease of BIBF 1120 trough plasma concentrations between TCs 1 and 2 when combined with docetaxel. Data indicate that BIBF 1120 mainly exhibited at least bi-exponential disposition kinetics.

The PK parameters for docetaxel were similar on day 1 of TCs 1 and 2 with little heterogeneity in terms of *t*_max_, gMean *t*_1/2_, gMean total body clearance, *C*_max_, and AUC_0−∞_ (data not shown). PK data of docetaxel in combination with BIBF 1120 are shown in Figure 1B.

### Response to therapy

Nineteen patients were evaluable for response using PSA values and/or dimensional RECIST criteria. BIBF 1120 treatment resulted in a disease response for 14 patients (73.7%), of which 13 (68.4%) showed a ⩾50% reduction in PSA levels; 1 of the 6 patients with measurable disease displayed partial response according to RECIST criteria. At the dose of 250 mg BID of BIBF 1120, the estimated progression-free survival rate at 24 weeks was 56%.

## Discussion

This phase I study was designed to evaluate the safety profile of an escalating dose of BIBF 1120 in combination with docetaxel given at the registered dose routinely used in this population. Overall, this combination was well tolerated, with preliminary signs of efficacy and no hint for PK interaction between BIBF 1120 and docetaxel.

As only 1 out of 12 patients experienced a DLT during the first TC at the dose of 250 mg BID, 250 mg of BIBF 1120 BID was the MTD. However, a more detailed analysis of the laboratory data revealed that CTCAE grade 3/4 increases in hepatic enzymes were observed in a total of six out of twelve patients (50%) at this dose level during the entire treatment period. Changes in the levels of transaminases might be primarily related to BIBF 1120 although the possible influence of docetaxel alone or additive effects by combining both compounds cannot be excluded. As in other phase I and II studies investigating BIBF 1120 monotherapy, these elevations were not associated with significant bilirubin elevation or prothrombin time modification and were fully reversible (Mross *et al*, 2010; Ledermann *et al*, 2011; Reck *et al*, 2011). The frequency of CTCAE grade 3/4 liver enzyme elevations was ∼20% in a phase II study investigating BIBF 1120 250 mg BID monotherapy in advanced lung cancer patients as compared with none at the 150-mg BID dose of BIBF 1120 (Reck *et al*, 2011). However, due to the small sample size in this phase I study, it is difficult to conclude whether the higher frequency of liver enzyme elevations is solely related to BIBF 1120 or to combination with docetaxel. With respect to other phase I studies investigating the combination of BIBF 1120 with either standard dose of pemetrexed (Ellis *et al*, 2010), paclitaxel, and carboplatin (du Bois *et al*, 2010) or FOLFOX (Prenen *et al*, 2010) in patients with NSCLC, gynaecological malignancies or colorectal cancer, respectively, the MTD of BIBF 1120 was also 200 mg BID. The combination regimens were shown to be well tolerated and were associated with an acceptable safety profile and no clinically relevant drug–drug interactions. Taking the overall safety profile of BIBF 1120 from other phase I, II mono and phase I combination studies into account, the recommended dose of BIBF 1120 was 200 mg BID for further investigations in this indication following the data analysis and discussions between the sponsor and the investigators at the end of the study.

In this study, other AEs observed were mild-to-moderate, allowing all patients to complete six courses of treatment with only three cases requiring dose reductions and sixteen patients continued treatment with BIBF 1120 monotherapy as part of a rollover study. During TC 1, the most frequent drug-related AEs were mostly gastrointestinal in nature. Moreover, BIBF 1120 does not significantly alter the toxicity profile of docetaxel; no increased frequency of neutropenia was observed with this combination in this study.

We found that the combination of BIBF 1120 and docetaxel offered promising activity with 68.4% of PSA responses. Moreover, seven patients (33.3%) experienced a complete biological response with a normalisation of PSA following treatment. In addition, one of the six patients with measurable disease had a partial response according to RECIST criteria. In the phase III studies of first-line metastatic HRPC, docetaxel monotherapy or docetaxel in combination with estramustine offered PSA responses of 45–50% (Petrylak *et al*, 2004; Tannock *et al*, 2004). The results observed in this trial can be at least partially attributed to BIBF 1120 inhibition of FGFR, VEGFR, and PDGFR (Hilberg *et al*, 2008). Several other molecules targeting VEGF/VEGFR have been tested in patients with HRPC with variable results (Stadler *et al*, 2004; Chi *et al*, 2008; Di Lorenzo *et al*, 2008). Bevacizumab, a monoclonal antibody targeting VEGF was evaluated in combination with docetaxel in docetaxel-pretreated patients and showed encouraging results of a >50% PSA response in 11 out of 20 patients in both responders and non-responders to docetaxel (Di Lorenzo *et al*, 2008). Sorafenib and sunitinib, two multi-kinase inhibitors targeting VEGFR2, gave less promising results, with PSA response rates of 3.6–12.1% (Chi *et al*, 2008; Dror Michaelson *et al*, 2009; Sonpavde *et al*, 2010). So far, no direct comparisons have been made to explain the differences in the effects of sunitinib, sorafenib, and other VEGF/VEGFR inhibitors in prostate cancer. It is likely that primary and acquired mechanisms of resistance to antiangiogenic therapies using VEGF/VEGFR inhibitors may occur early in some patients with advanced prostate cancer. In several preclinical models, FGFR activation has been proposed as a mechanism that compensates for VEGFR inhibition in resistant tumours (Bergers and Hanahan, 2008). The wide spectrum of kinase activity observed with BIBF 1120, including FGFR inhibition, may be considered as a potential advantage to prevent or delay resistance due to VEGFR/PDGFR inhibition in human tumours.

This study included a dose-dependent increase in exposure with increasing doses of BIBF 1120, and detected high interpatient variability in *C*_max_ and AUC. BIBF 1120 exhibited at least bi-exponential disposition kinetics, and differences in plasma concentration–time profiles between patients were predominantly attributed to differences in time to reach the *C*_max_ (*t*_max_) and not due to differences in distribution or elimination (data not shown). PK data of docetaxel in combination with BIBF 1120 were similar to that expected with docetaxel alone (Bruno *et al*, 1996). The PK parameters outlined in the phase I study of BIBF 1120 monotherapy included a high drug clearance, a *t*_1/2_ of ∼15 h and plasma concentrations reaching their maximum after ∼3 h of drug intake. All of these parameters are consistent with the *t*_1/2_ reported in TC 2 of this study (Mross *et al*, 2010).

In summary, BIBF 1120 200 mg BID in combination with docetaxel and prednisone is the recommended dose for patients with metastatic HRPC. This combination was well tolerated, with preliminary efficacy and no indication of PK interaction. Angiogenesis in prostate cancer appears as a complex process that involves several growth factor receptors. Identification of biomarkers that may predict effects of multi-kinase inhibitors such as BIBF 1120 may be of great help in the future development of such drugs in hormone-resistant prostate cancer patients.

## Figures and Tables

**Table 1:** Patient characteristics

**Number of patients evaluable**	**BIBF 1120 250 mg BID (*n*=12)**	**All patients (*n*=21)**
Median age, years (range)	67.5 (58–79)	68 (58–79)
Median time between metastatic diagnosis and inclusion in the study, months (range)	3.6 (0.9–70.6)	2.7 (0.5–70.6)
		
*Number of metastatic sites, n (%)*		
0	1 (8.3)	2 (9.5)
1	7 (58.3)	12 (57.1)
⩾2	4 (33.3)	7 (33.3)
		
*Location of metastatic sites, n (%)*		
Bone	6 (50)	10 (47.6)
Bone and lymph nodes	4 (33.3)	4 (19)
Lymph nodes	1 (8.3)	3 (14.3)
		
*WHO performance status, n (%)*		
0	10 (83.3)	16 (76.2)
1	2 (16.7)	5 (23.8)
		
Mean initial PSA, range (ng ml^−1^)	162.7 (9–1521)	108.5 (3–1521)
Prior prostate/prostatic region radiotherapy, *n* (%)	5 (41.7)	12 (57.1)

Abbreviations: WHO=World Health Organization; PSA=prostrate serum antigen.

**Table 2:** Frequency of patients with drug-related AEs (reported in >1 patient or of CTCAE grade 3/4) during all courses

	**BIBF 1120 100 mg BID (*n*=3)**		**BIBF 1120 150 mg BID (*n*=3)**		**BIBF 1120 200 mg BID (*n*=3)**		**BIBF 1120 250 mg BID (*n*=12)**		**Total (*n*=21)**	
**Preferred term**	**All**	**Gr 3/4**	**All**	**Gr 3/4**	**All**	**Gr 3/4**	**All**	**Gr 3/4**	**All**	**Gr 3/4**
Asthenia	0	0	3	0	1	0	10	0	14	0
Diarrhoea	0	0	3	0	2	2	8	0	13	2
Nausea	0	0	2	0	2	0	5	0	9	0
Anorexia	0	0	3	0	0	0	3	0	6	0
Epistasis	0	0	1	0	1	0	3	0	5	0
Vomiting	0	0	0	0	2	0	3	0	5	0
Dysphonia	0	0	1	0	2	0	1	0	4	0
Upper abdominal pain	0	0	1	0	0	0	3	0	4	0
Alopecia	0	0	0	0	0	0	4	0	4	0
Elevated ALT	0	0	0	0	0	0	4	2	4	2
Elevated AST	0	0	0	0	0	0	3	2	3	2
Dysgeusia	0	0	1	0	0	0	2	0	3	0
Constipation	0	0	1	0	0	0	2	0	3	0
Elevated GGT	0	0	0	0	0	0	3	2	3	2
Pyrexia	0	0	1	0	0	0	1	0	2	0
Ageusia	0	0	1	0	0	0	1	0	2	0
Headache	0	0	2	0	0	0	0	0	2	0
Neuropathy	0	0	1	0	0	0	1	0	2	0
Hot flush	0	0	0	0	1	0	1	0	2	0
Dyspepsia	0	0	1	0	0	0	1	0	2	0
Rash	0	0	0	0	0	0	2	0	2	0
Muscle spasm	0	0	0	0	0	0	2	0	2	0
Mucosal inflammation	0	0	0	0	0	0	2	0	2	0
Weight decrease	0	0	1	0	0	0	1	0	2	0
Elevated alkaline phosphatase	0	0	0	0	0	0	2	0	2	0

Abbreviations: AE=adverse event; CTCAE=Common Terminology Criteria for Adverse Events; Gr=grade; ALT=alanine aminotransferase; AST=aspartate aminotransferase; GGT=γ-glutamyl transpeptidase.

**Table 3:** Pharmacokinetic parameters of BIBF 1120 following oral administration of 250 mg BID

	**Treatment cycle 1**		**Treatment cycle 2**	
	***n***	**gMean (gCV%)**	***n***	**gMean (gCV%)**
*C*_max_ (ng ml^−1^)	11	65.0 (107)	11	45.8 (98.5)
AUC_0−24_ (ng h ml^−1^)	11	454 (85.3)	10	410 (65.5)
*t*_1/2_ (hours)	10	7.03 (29.2)	9	15.4 (88.1)
*t*_max_ (hours)^a^	11	3.00 (0.9–7)	11	2.08 (0–7)

Abbreviations: gMean=geometric mean; gCV=geometric coefficient of variation; *C*_max_=plasmatic peak concentrations following the first dose; AUC=area under the curve; *t*_1/2_=half-life time; *t*_max_=time from dosing to peak concentration.

a Median and range.

**Table 4:** Pharmacokinetic parameters of docetaxel following treatment with 75 mg m^−2^ and BIBF 1120 250 mg BID

	**Treatment cycle 1 (*n*=12)**	**Treatment cycle 2 (*n*=12)**
	**gMean (gCV%)**	**gMean (gCV%)**
*C*_max_ (ng ml^−1^)	1510 (33.7)	1980 (94.5)
AUC_0−∞_ (ng h ml^−1^)	1860 (34.6)	2460 (61.9)
*t*_1/2_ (hours)	20.2 (16.8)	19.4 (11)
*t*_max_ (hours)^a^	1.02 (0.8–1.3)	1.01 (1–1.5)

Abbreviations: gMean=geometric mean; gCV=geometric coefficient of variation; *C*_max_=plasmatic peak concentrations following the first dose; AUC=area under the curve; *t*_1/2_=half-life time; *t*_max_=time from dosing to peak concentration.

a Median and range.
